# The Functional Role of Long Non-Coding RNAs in Melanoma

**DOI:** 10.3390/cancers13194848

**Published:** 2021-09-28

**Authors:** Michal Wozniak, Malgorzata Czyz

**Affiliations:** Department of Molecular Biology of Cancer, Medical University of Lodz, 92-215 Lodz, Poland; malgorzata.czyz@umed.lodz.pl

**Keywords:** melanoma, lncRNA, drug resistance, targeted therapy, oncogenes, tumor suppressors, gene regulation, miRNA sponging

## Abstract

**Simple Summary:**

Long non-coding RNAs (lncRNAs) are heterogeneous RNA molecules that can regulate a plethora of cellular processes including proliferation, differentiation, migration, invasion and apoptosis through diverse mechanisms. Growing evidence indicates that aberrant expression of lncRNA leads to the development and progression of cancer, including melanoma, and might contribute to the acquisition of drug resistance. Moreover, their tightly-regulated expression and their high tissue and disease specificity make them promising biomarkers in non-invasive diagnostics of cancer. This review summarizes current knowledge regarding the involvement of lncRNAs in melanoma along with their possible use as diagnostic biomarkers.

**Abstract:**

Melanoma is the most lethal skin cancer, with increasing incidence worldwide. The molecular events that drive melanoma development and progression have been extensively studied, resulting in significant improvements in diagnostics and therapeutic approaches. However, a high drug resistance to targeted therapies and adverse effects of immunotherapies are still a major challenge in melanoma treatment. Therefore, the elucidation of molecular mechanisms of melanomagenesis and cancer response to treatment is of great importance. Recently, many studies have revealed the close association of long noncoding RNAs (lncRNAs) with the development of many cancers, including melanoma. These RNA molecules are able to regulate a plethora of crucial cellular processes including proliferation, differentiation, migration, invasion and apoptosis through diverse mechanisms, and even slight dysregulation of their expression may lead to tumorigenesis. lncRNAs are able to bind to protein complexes, DNA and RNAs, affecting their stability, activity, and localization. They can also regulate gene expression in the nucleus. Several functions of lncRNAs are context-dependent. This review summarizes current knowledge regarding the involvement of lncRNAs in melanoma. Their possible role as prognostic markers of melanoma response to treatment and in resistance to therapy is also discussed

## 1. Introduction

Melanoma is the most lethal form of melanocyte-originated skin cancer and its incidence continues to increase worldwide [1,2]. While in the early stage, melanoma can be safely treated with surgery alone, the metastatic melanoma is highly refractory to cytotoxic therapy. Given the well-defined pathogenic role of mutated BRAF^V600^ in melanoma, targeting this kinase and its substrate MEK1/2 has been in focus of several studies and clinical trials resulting in FDA-approved inhibitors such as vemurafenib, dabrafenib and encorafenib to block enhanced activity of mutated BRAF, and trametinib, binimetinib and cobimetinib to inhibit the activity of MEK1/2 [3,4,5]. Moreover, combinations of BRAF and MEK inhibitors have shown improved patient outcomes [6,7]. In parallel to targeted therapies, immune checkpoint inhibitors (ICIs), anti-CTLA4 antibodies (ipilimumab) and anti-PD1/PDL1 antibodies (pembrolizumab, nivolumab, atezolizumab) have been successfully developed showing tumor regression and long-term durable disease control in melanoma patients [8,9]. Combined inhibition of CTLA4 and PD1 has high activity against brain metastasis and the highest 5-year overall survival rate of all therapies used in advanced melanomas, although immune-related adverse events occur frequently [10]. Combinations of ICIs with targeted or oncolytic therapies are also under investigation [11,12,13,14,15]. While the clinical use of the BRAF^V600^ and immune checkpoint inhibitors improved the prospects of melanoma patients, death rates still remain unsatisfactory high, and several questions about melanoma diagnosis, development of metastasis, lack of response and resistance to immune- and targeted- therapy remain to be addressed [16,17,18]. Specific and highly sensitive biomarkers of response to either targeted therapy or immune checkpoint inhibitors are still needed.

Large scale genomic-wide studies revealed that only about 1–2% of human genome codes for proteins [19], and approximately 90% of genome is transcribed into RNA, including two main regulatory types of non-coding RNAs: microRNAs (miRNAs) and long non-coding RNAs (lncRNAs) [20]. miRNAs are linked to the regulation of many cellular processes, such as proliferation, differentiation, senescence, survival, autophagy, and migration [21,22,23]. Evidence accumulated during the last decade indicates that lncRNAs are also involved in the regulation of the same processes. lncRNA molecules are usually 200–1000 bases long. Their genes are characterized by the same histone modifications as protein-coding genes and are transcribed by RNA polymerase II from independent promoters. lncRNA transcripts are usually 5’-capped and 3′-polyadenylated, often spliced with similar exon/intron lengths as mRNAs [19,24,25,26]. lncRNAs expression is tightly regulated and is much more tissue- and disease-specific than mRNAs [27], therefore, lncRNAs might be used as potential specific markers in non-invasive diagnostics of diseases such as cancer, even though they are present at 10–100-times lower levels than the mRNAs [19]. Despite low overall sequence similarity, lncRNAs possess evolutionarily-conserved roles and secondary structure with regions of microhomology important for their functions [28,29]. Depending on cellular localization, lncRNAs may control and modulate crucial signaling pathways in human cells: nuclear lncRNAs, such as DIRC, FALEC, PVT1 or HEIH, are involved in chromatin modification providing epigenetic marks, transcriptional regulation, and RNA processing, while cytoplasmic lncRNAs (TLSCN, MIRAT or SAMMSON) modulate mRNA stability and translation, and control various cellular signaling cascades [30]. It has been demonstrated that lncRNAs exert limited potential to code for proteins [31,32]. lncRNAs can directly interact with proteins influencing their stability, activity and localization [33], act as sponges for miRNAs, reducing their regulatory effect on targeted mRNAs [34], or even serve as precursors of miRNAs or circular (circ)RNAs [30]. lncRNAs play an important role as a determinant of drug resistance in several cancers such as lung, ovarian, gastric and breast cancers or melanoma [35]. The involvement of lncRNAs in uveal melanoma has been recently reviewed [36,37].

Many lncRNA genes are located in the genomic regions that are frequently deleted or amplified in cancer cells [38]. Therefore, low expression of lncRNA with tumor suppressor properties or upregulation of oncogenic lncRNAs may significantly contribute to development of various types of cancer, including melanoma. Moreover, recent advances in high throughput RNA sequencing (RNA-seq) methods have allowed to identify novel non-coding RNAs, as well as new splicing isoforms of the previously classified ones [39]. A comprehensive study of Iyer et al. identified more than 58,000 mature lncRNAs from 7256 RNA libraries derived from human tissues, including 5298 primary cancers and 281 metastases [40]. Among them, 7941 lncRNAs were either cancer- or lineage-specific, and 339 lncRNAs were associated with melanoma. Moreover, the authors have identified nearly 8000 lineage- or cancer-specific lncRNAs that might serve as disease biomarkers. 

This review summarizes current knowledge regarding the involvement of lncRNAs in the development and progression of cutaneous melanoma. We begin by describing the protein binding property of lncRNAs, primarily focusing on direct interaction with histone modifiers and transcriptional modulators in nucleus, followed by miRNA sponging activities of lncRNA that modulate mRNA turnover in the cell. Finally, we highlight the potential use of selected lncRNA as biomarkers of the treatment responses and drug resistance developed in melanoma patients.

## 2. Protein-lncRNA Interactions in Melanoma

lncRNAs are able to bind to various proteins through specific RNA-binding domains. It has been suggested that lncRNAs serve as protein scaffolds forming ribonucleoprotein (RNP) complexes that bring proteins in proximity. Ribeiro et al., have predicted that 847 lncRNAs (which constitutes approximately 5% of the whole long non-coding transcriptome) may bind to about half of known interacting proteins [41]. lncRNAs that specifically bind to one target protein can be found, however, most of lncRNAs bind to a vast number of different proteins and the protein binding affinity is highly tissue- and context -dependent. lncRNA-protein interactions have two main roles in melanoma: direct regulation of gene transcription in the nucleus and fine-tune protein stability and activity in the cytoplasm (Table 1).

### 2.1. lncRNAs Regulating Transcription in Nucleus

**Figure 1:**
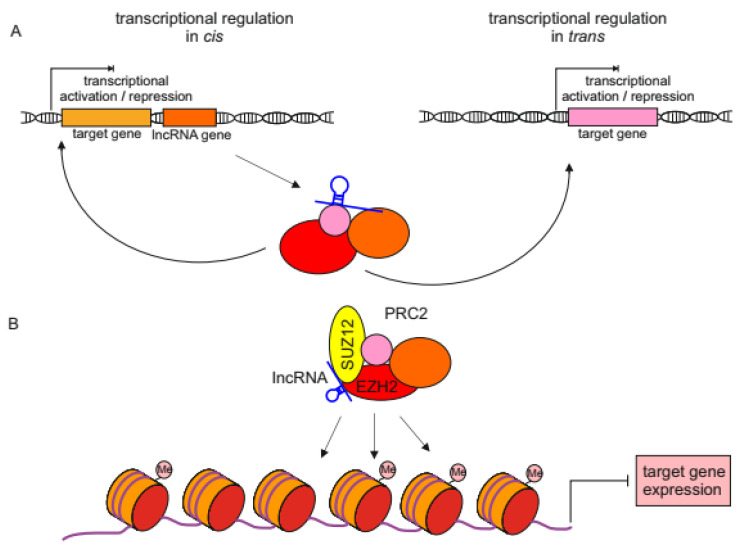
The mechanisms of transcription regulation mediated by lncRNAs in the nucleus. (**A**) lncRNA bind to the effector protein complex that activates or represses the expression of target genes. lncRNAs may act in cis, where they regulate the expression of adjacent or proximal genes on the same chromosome, or in trans, regulating distant genes from other chromosomes. (**B**) lncRNAs bind to polycomb repressive complex 2 (PRC2) mostly through EZH2 or SUZ12 protein subunits. This complex facilitates histone 3 lysine 27 (H3K27me3) trimethylation and occupancy of target gene promoter which results in target gene repression.

Numerous types of lncRNAs reside in the nucleus where they function mostly as regulators of expression by forming complexes with effector proteins (histone modifiers, transcription factors, etc.) thus facilitating their attachment to the promoter of a target gene. lncRNAs can act in cis, where they regulate the expression of adjacent or proximal genes on the same chromosome, or in trans, influencing distant genes on different chromosomes [60] (Figure 1A). Table 1 shows that in melanoma cells most of lncRNAs in the nucleus act in trans. Taking into consideration generally low expression of lncRNAs, even with one to few molecules per cell [19,29], an in cis mechanism of action should be privileged, as transport to distant chromosomes would make lncRNAs acting in trans less potent to mediate regulation of gene expression.

**Figure 2:**
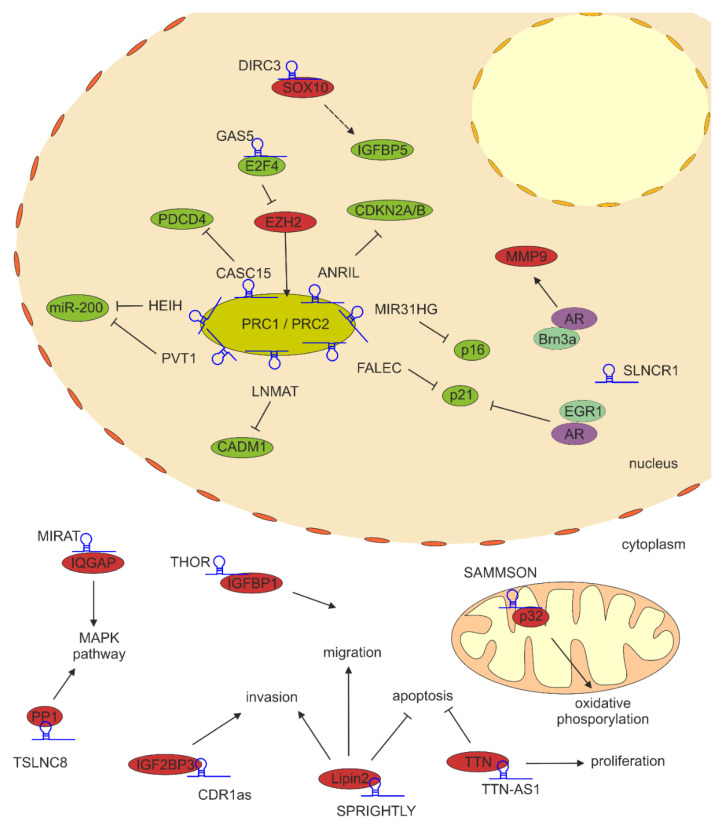
lncRNA-protein interactions in melanoma cells. The mechanism of action and function of various lncRNAs is predominantly imposed by their subcellular localization. lncRNAs can reside in the nucleus, where they bind to activators or repressors of transcription, as well as to transcription factors, thereby regulating the expression of target genes. Cytosolic lncRNAs modulate signaling pathways that control melanoma cell proliferation, invasion and migration. Mitochondrial lncRNAs control oxidative phosphorylation and sensitivity to apoptosis. Dashed arrow indicates indirect activation of expression.

In terms of tumorigenesis, lncRNAs frequently bind to a polycomb repressive complexes, PRC1 and PRC2. These complexes transfer chromatin repressive modifications on histone tails and occupy promoters of repressed genes. The most frequent binding partner of various lncRNAs within the PRC2 complex is Enhancer of Zeste 2 (EZH2) protein, a histone methyltransferase that trimethylates lysine 27 on histone 3 (H3K27me3), which is a marker of transcriptionally silent chromatin. [61] (Figure 1B). Moreover, this protein is frequently overexpressed in numerous solid tumors, which leads to tumor progression and increased invasiveness [62] (Figure 2; Table 1).

In melanoma cells, PRC1 and PRC2 negatively regulate oncogene-induced senescence (OIS). OIS, first reported by Serrano et al. [63], is a senescence process triggered by hyperactive oncogenes such as RAS or BRAF and is considered to be a barrier against tumorigenesis. Senescent cells are present in pre-malignant tissues but absent in tumor cells, indicating that senescence must be bypassed by additional alterations to progress towards malignancy [64]. Senescence is mediated by two pathways controlled by p14^ARF^ and p16^INK4A^ and both tumor suppressors are encoded by the same locus *INK4B-ARF-INK4A* on chromosome 9p.21. During senescence, PRC1 and PRC2 complexes, that suppress the expression of *INK4B-ARF-INK4A*, are dissociated from the chromatin, which results in transcriptional activation of both genes [65]. MIR31HG and ANRIL are two lncRNAs that regulate this genomic locus through interactions with PRC1 and PRC2 in melanoma. miR-31 host gene (MIR31HG) lncRNA is upregulated in OIS upon BRAF overexpression, binds directly to SUZ12 polycomb repressive complex 2 subunit and EZH2, thus is required for the repression of the *INK4A* locus, whereas knockdown of MIR31HG promotes a p16^INK4A^-dependent senescence of melanoma cells [50]. 

Another lncRNA ANRIL (antisense non-coding RNA in the *INK4A* locus) has been originally identified in familial melanoma patients with a large germline deletion in the *INK4B-ARF-INK4A* gene cluster [66] and has been found to be dysregulated in several other cancers, such as gastric or breast cancers [67,68]. The most commonly accepted function of ANRIL is to mediate repression of the CDKN2A/B locus by association with PRC1 through direct binding of chromobox 7 (CBX7) protein [42]. In addition, ANRIL silencing activates the expression of both p14^ARF^ and p16^INK4A^ tumor suppressors [69]. However, recent identification and characterization of differently-spliced isoforms of ANRIL in melanoma, including its covalently-closed circular form, adds another level of complexity to this regulatory network [70].

Other lncRNAs that contribute to gene repression in melanoma through direct interaction with EZH2 are CASC5, FALEC, HEIH, LNMAT1 and PVT1. Chromosome 6p22.3 cancer susceptibility candidate 15 (CASC15) long intergenic non-coding RNA (lincRNA) locus has been identified as frequently amplified genomic segment in metastatic melanoma tumors and cell lines [71]. Indeed, CASC15 is upregulated in melanoma tissues when compared to control samples, and its high expression has been associated with tumor, nodes, metastasis (TNM) staging, and distal and lymphatic metastasis [43]. CASC15 knockdown inhibits proliferation, facilitates apoptosis and suppresses invasion. CASC15-EZH2 complex silences the expression of programmed cell death 4 (PDCD4) tumor suppressor gene [43].

Focally amplified long non-coding RNA in epithelial cancer (FALEC) is overexpressed in melanoma tissues and cell lines, when compared to respective healthy controls and its higher expression has been linked to poor patients’ survival, and TNM staging [46]. Knockdown of FALEC blocks invasion and epithelial-to-mesenchymal transition (EMT)-like process, and induces the expression of p21, an inhibitor of cyclin-dependent kinases. FALEC predominantly resides in the nucleus, where it binds to EZH2, and this interaction in melanoma facilitates binding to p21 promoter and increases H3K27me3 modification, therefore repressing p21 transcription [46].

High expression in hepatocellular carcinoma (HEIH) lncRNA, first identified as an oncogene in liver cancers [72] has been also upregulated in melanoma cell lines and tissues. High levels of HEIH are associated with advanced clinical stages and might predict poor clinical outcomes in melanoma patients [48]. The HEIH-EZH2 RNA-protein complex binds to the promoter of miR-200 family genes, which results in repression of miR-200a, miR-200b and miR-429 [48]. The members of miR-200 family are well-known tumor suppressors that modulate cell proliferation, migration, invasion and drug resistance in various cancers, including melanoma [73]. Another lncRNA that represses miR-200 family expression through EZH2-mediated binding to miR-200 promoter is plasmacytoma variant translocation 1 (PVT1), also upregulated in melanoma. PVT1 silencing significantly inhibits cell proliferation, migration and invasion, and induces cell cycle arrest which is manifested by decreased cyclin D, N-cadherin and vimentin, and increased E-cadherin expression [52]. Finally, the lymph node metastasis associated transcript 1 (LNMAT1) is a key regulator in lymph node metastasis of several cancers and its oncogenic role has been also established in melanoma. Upregulated LNMAT1 binds and recruits EZH2 to the cell adhesion molecule 1 (CADM1) promoter, silencing the expression of this tumor suppressor. LNMAT knockdown results in decreased matrix metalloproteinases MMP2 and MMP9 expression and inhibits invasion and migration of melanoma cells [49].

EZH2 expression itself can be also regulated by lncRNAs. Growth arrest-specific transcript 5 (GAS5) lncRNA recruits E2F transcription factor 4 (E2F4) to EZH2 promoter, which results in repression of EZH2 transcription. Attenuated EZH2 expression rescues EZH2-mediated cyclin dependent kinase inhibitor 1C (CDKN1C) downregulation which in turn promotes oxidative stress and apoptosis [47]. Moreover, GAS5 activates transcription of miR-137 tumor suppressor [74]. GAS5 low expression has been correlated with the TNM staging of melanoma patients [74].

Long non-coding RNAs can also bind to other transcription factors and modifiers and activate transcription. SRA-like non-coding RNA 1 (SLNCR1) gene is located on chromosome 17 in a region frequently amplified in melanoma and lung and ovarian cancers [54]. Its high expression has been associated with shorter overall survival (OS) in melanoma patients, compared to patients with low SLNCR1 expression. SLNCR1 contains highly conserved sequence of approx. 300 nt that shows extensive similarity to steroid receptor RNA activator 1 (SRA1) sequence, hence the name of this lncRNA. Indeed, SLNCR1 can bind to androgen receptor (AR) within this sequence, and to brain-specific homeobox protein 3a (Brn3a) with sequence adjacent to the steroid-responsive element. This complex in turn binds to MMP9 promoter, activating the expression of matrix metalloproteinase 9, thus promoting melanoma cell invasion [54]. Since AR-mediated transcription program has been linked to tumorigenesis [75], the SLNCR1-mediated increase in AR-dependent transcriptional activity in melanoma might explain higher metastatic rate and lower OS of male melanoma patients. On the other hand, SLNCR1 can also mediate gene repression: SLNCR1 recruits AR directly to EGR1-bound chromatin, where it acts as a transcription switch and represses EGR1-inducible p21 expression, therefore promoting proliferation of melanoma cells [55]. Taking into consideration the involvement of SLNCR1 in the regulation of transcription program of melanoma, blocking the AR-SLNCR1 interaction with specific oligonucleotides might be an attractive therapeutic strategy in the future [76].

Disrupted in Renal Carcinoma 3 (DIRC3) lncRNA is transcriptionally repressed by microphthalmia-associated transcription factor (MITF)- SRY-box transcription factor 10 (SOX10) pathway in melanoma. DIRC3 acts in cis and modifies chromatin structure and suppresses SOX10 binding to the promoter of neighboring insulin-like growth factor binding protein 5 (IGFBP5) gene, which activates this tumor suppressor and inhibits anchorage-independent melanoma cell growth [45]. Moreover, IGFBP5 negatively regulates IGFR1 and MAPK kinase signaling, which results in the inhibition of proliferation and metastasis [77]. Since DIRC3 expression is negatively correlated with both MITF and SOX10 levels, its expression increases in invasive MITF^low^ melanoma cells. Indeed, TCGA data analyzed by Coe et al., indicate that DIRC3 level correlates with a gene expression profile of invasive melanoma [45]. Therefore, DIRC3 may have dual roles at different stages of the disease and down-regulation of DIRC3 tumor suppressor function in metastatic melanomas may be an important event in melanoma progression. Phenotype switching between different proliferative (MITF^high^) and invasive (MITF^low^) states is accompanied by large scale changes in chromatin structure [78], therefore, DIRC3 might be one of the factors regulating proliferative or invasive chromatin imprinting.

### 2.2. lncRNA Scaffolding Proteins in Melanoma

Many lncRNAs have been identified in human cells that bind to various proteins and affect their stability and activity. Dysregulated expression of these lncRNAs might lead to cancer development and progression. The most prominent lncRNA involved in melanomagenesis are: SPRIGHTLY, SAMMSON, MIRAT, THOR, TSLNC8, TTN-AS1 and circular RNA CDR1as.

SPRIGHTLY (also known as SPRY4-IT1) is one of the first lncRNAs identified in melanocytes and melanoma, and is consistently upregulated in samples from melanoma patients, when compared to control melanocytes. Elevated SPRIGHTLY level has been also found in plasma of melanoma patients and is closely related with tumor stage. Elevated SPRIGHTLY significantly reduces OS rates of patients and might be considered as an independent prognostic factor in patients with melanoma [79]. SPRIGHTLY is predominantly localized in the cytoplasm [80], where it regulates lipid metabolism by directly binding to lipin 2 enzyme, that converts phosphatidates to diacylglycerols. SPRIGHTLY knockdown might induce apoptosis through alterations in lipid metabolism, that leads to cellular lipotoxicity [56]. Moreover, downregulation of SPRIGHTLY in melanoma cells significantly reduces proliferation, migration and invasion [80].

Survival-Associated Mitochondrial Melanoma Specific Oncogenic Non-Coding RNA (SAMMSON) is a melanoma-specific lncRNA and its expression is positively regulated by SOX10, a transcription factor responsible for the development of neural and pigment cells, that derive from the neural crest. SAMMSON gene is located ~30 kb downstream of MITF and both genes are co-amplified in about 10% of melanomas [53], and amplification of both genes is associated with poor prognosis [81]. SAMMSON expression was comparable in proliferative and invasive melanoma phenotypes, whereas MITF-M protein level was high in the proliferative and low in the invasive cultures, respectively. SAMMSON localizes mostly in cytoplasm, with a subset of molecules localized in mitochondria. p32, a protein required for the maintenance of mitochondrial membrane potential and oxidative phosphorylation [82,83,84] has been identified as a binding partner for SAMMSON. p32 level is elevated in various cancers, including melanoma, and its knockdown decreases tumor growth [82,85]. The silencing of SAMMSON expression reduces the mitochondrial localization of p32, thereby disrupting its vital mitochondrial functions, although the total p32 level remains unaffected. Reduction of mitochondrial p32 levels or SAMMSON knockdown perturbs mitochondrial respiratory chain and collapses the mitochondrial membrane potential, which eventually leads to mitochondria-dependent apoptosis of melanoma cells [53].

CDR1as is an atypical circular (circ) RNA molecule that arises antisense to a putative protein-coding gene, but without any known linear counterpart from the same strand [86,87]. Loss of CDR1as expression has been identified in panels of primary and metastatic melanoma cell lines [44,88] and this loss is associated with shorter progression-free and overall survival of melanoma patients [44]. Although CDR1as has been primarily thought to negatively regulate miR-7 acting as miRNA sponge in neuronal cells [86,87], Hanniford et al. [44] failed to detect changes in miR-7 activity after CDR1as depletion and inhibition of miR-7 did not revert the effects of CDR1as depletion on melanoma cell invasion, providing evidence that miR-7 deregulation is not a critical mediator of CDR1as silencing effects in melanoma. Instead, the authors points at direct interactions with a specific RNA-binding protein (RBP) insulin-like growth factor 2 mRNA-binding protein 3 (IGF2BP3), and CDR1as depletion in melanoma triggers IGF2BP3-mediated upregulation of SNAI2 and MEF2C that positively regulate invasion and transcription of neural crest genes, respectively [44].

Testis Associated Oncogenic lncRNA (THOR) is an evolutionarily conserved lncRNA that is frequently upregulated in several cancers, including melanoma [57]. THOR directly binds to and stabilizes insulin-like growth factor binding protein 1 IGF2BP1, an RNA-binding protein, that in turn increases the stability of its mRNA targets. Melanoma model in zebrafish has confirmed that THOR-IGF2BP1 interaction promotes melanoma development and progression by stabilizing several oncogenes such as IGF2 and CD44 [57].

lncRNA titin antisense 1 (TTN-AS1) is transcribed in the opposite direction of the human titin (TTN) gene. This lncRNA is highly expressed in the panel of melanoma samples in TCGA skin cutaneous melanoma (SKCM) dataset, and high expression of this lncRNA is correlated with poor OS of melanoma patients [59]. Additionally, RNA pull-down and LC/MS-MS experiments performed by Wang, et al. have identified 179 proteins (among them vimentin or fibronectin) that directly interact with TTN-AS1. These proteins participate in RNA binding and cytoskeletal protein binding or are involved in antioxidant activity. However, the interactions between TTN-AS1 and identified proteins have not been further studied in melanoma [59]. Increased expression of TTN-AS1 results in significant upregulation of TTN at mRNA and protein levels, and high expression of both genes induces melanoma cell proliferation, suppresses cell apoptosis, promotes cell migration in vitro and tumor growth and metastasis in vivo. Mechanistically, TTN-AS1 binds to and enhances transcription of TTN gene in the nucleus and stabilizes its mRNA in the cytoplasm. Deficiency of both TTN and TTN-AS1 triggered apoptosis, cell cycle arrest and decrease in expression of cyclin D1, CDK2 and CDK4 in melanoma cells [59].

Tumor suppressor lncRNA on chromosome 8p12 (TSLNC8) gene is located on 8p12 locus, characterized with frequent loss of heterozygosity in hepatocellular carcinoma (HCC) tissues [89]. TSLNC8 binds to PP1α protein, one of three catalytic subunits of protein phosphatase 1 (PP1). This subunit dephosphorylates BRAF inhibitory phosphorylation sites [90]. Drug-mediated downregulation of TSLNC8 triggers PP1α accumulation in cytoplasm which results in reactivation of MAPK signaling [58].

MAPK Inhibitor Resistance Associated Transcript (MIRAT), a cytoplasmic intergenic lncRNA, functions by binding to the scaffold protein IQ motif containing GTPase activating protein 1 IQGAP1 [51], a protein facilitating MEK/ERK pathway signaling. MIRAT stabilizes IQGAP1 and inhibits its decay [91,92].

## 3. Competing Endogenous RNAs in Direct interactions with miRNAs

**Figure 3:**
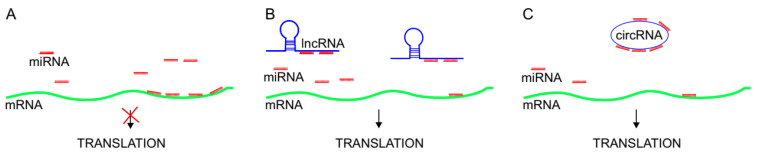
The mechanism of sponging activity of lncRNAs and circRNAs. (**A**) In the absence of lncRNAs, abundant miRNAs bind to the 3′ untranslated region of target transcripts and block their translation. (**B**,**C**). lncRNAs or circRNAs, when present, outcompete target transcripts in miRNA binding, which results in the rescue of translation blockade of target transcripts.

lncRNAs that predominantly reside in the cytoplasm exert the ability to bind miRNAs according to their regions of sequence complementarity, therefore they are termed competing endogenous RNAs (ceRNA), or RNA sponges [93]. Two types of direct miRNA-lncRNA interactions have been identified so far, miRNA-mediated lncRNA decay and lncRNA acting as decoy molecules for miRNAs [94]. The latter has been reported to be the most prevalent interaction impacting cancer development and progression. Such interaction results in miRNA binding to lncRNA 3’ end through miRNA response elements (MRE) with complete or partial sequence complementarity with respective miRNA. As a result, the translation of bona fide miRNA coding target transcripts is promoted (Figure 3A,B). Therefore, lncRNAs serving as miRNA sponges act as positive regulators of miRNA target transcript translation [93,95]. In this regard, lncRNAs may be considered as tumor-promoting (e.g., BANCR), when their sponging activity triggers the increase of oncogenes expression, or tumor suppressors (e.g., MEG3), when they positively regulate the expression of tumor suppressor genes [96].

The miRNA sponging property is typical not only for classic (5′-capped and 3′-polyadenylated) lncRNAs, but also for circRNAs [86,87] (Figure 3C). circRNAs are functional non-coding RNAs that are composed of single-stranded, covalently-closed circular transcripts resistant to nuclease-mediated degradation [97]. Previously considered to be only a by-product of aberrant RNA splicing [98,99], circRNAs are now regarded as ubiquitously and sometimes abundantly expressed endogenous molecules, highly evolutionary-conserved among mammals [100,101,102]. Apart from functioning as miRNA sponges and regulating proliferation, differentiation, and invasion of cancer cells [103], other molecular functions of circRNAs embrace sequestering RNA binding proteins (RBPs) [104,105] and possible translation into proteins [106]. Both types of competing endogenous RNA molecules (lncRNAs and circRNAs) are actively involved in melanomagenesis, and their sponging activity is summarized in Table 2.

### 3.1. lncRNAs as miRNA Sponges in Melanoma

The thorough bioinformatics analysis of public melanoma datasets performed by Zhu et al., [152] have identified three lncRNAs, metastasis associated lung adenocarcinoma transcript 1 (MALAT1), LINC00943 and LINC00261 that might be considered key lncRNAs involved in melanoma development and progression because: (I) all three lncRNAs control the highest number of lncRNA-miRNA and miRNA-mRNA interactions, (II) they are significantly overexpressed in melanoma tissues compared to healthy tissue and (III) their expression has been identified as prognostic markers and potential therapeutic targets in melanoma. MALAT1 is characterized with high potential for sponging numerous miRNAs such as miR-34a [133], miR-183 [134] or miR-22 [135]. miR-34a is a p53-inducible tumor suppressor [153] that is able to bind and inhibit various oncogenes, e.g., cyclin D1, CREB or CDK4/6 in different tumors, such as prostate and colorectal cancer or myeloid leukemia [154]. In melanoma, miR-34a targets c-Myc and Met transcripts [133]. miR-183, downregulated in melanoma, targets integrin subunit beta 1 (ITGB1) which is associated with tumor growth, metastasis and drug resistance [134], and miR-22 inhibits the expression of EMT-promoting MMP14 and Snail transcription factors [135]. Overexpressed MALAT1 sponges these three miRNAs which leads to the upregulation of aforementioned oncogenes in melanoma [133,134,135]. MALAT1 has been initially identified to be upregulated in melanoma, together with UCA1 lncRNA, by Tian et al. [155]. The expression of urothelial cancer associated 1 (UCA1) has been positively correlated with melanoma stage and, although not statistically significant, with metastatic status. Mechanistically, UCA1 binds and inhibits the activity of miR-28 and miR-507 which leads to upregulation of tumor-promoting homeobox B3 (HOXB3), and forkhead box M1 (FOXM1) transcription factors, respectively [147,148].

One of several lncRNAs upregulated in BRAF^V600E^ melanomas is BRAF-activated non-coding RNA (BANCR), a mostly melanoma/melanocyte-specific RNA molecule. The expression of BANCR has been positively correlated with melanoma stage, and patients with high BANCR expression are characterized with lower OS [156]. Downregulation of BANCR in BRAF-mutated melanoma cell lines alters the expression of 88 genes, and most of the repressed genes have been involved in melanoma motility. One of them, the C-X-C motif chemokine ligand 11 (CXCL11), is able to reverse melanoma cells motility, when upregulated in BANCR-depleted cells [157]. Moreover, BANCR downregulation significantly decreases melanoma cells proliferation in vitro and tumor size and weight in vivo, mostly via deactivation of ERK and JNK signaling. This effect is synergistically increased when BANCR downregulation has been combined with ERK and JNK inhibitors UO126 and SP600125, respectively [156]. BANCR has the ability to sponge miR-204-5p [107]. The expression of miR-204-5p is lost in melanoma tissues and cell lines and this loss has been correlated with worse OS of melanoma patients [158,159]. BANCR-mediated miR-204-5p sponging activates Notch2, a direct target of this miRNA, which is highly expressed in metastatic melanoma [160].

Colon cancer-associated transcript 1 (CCAT1) is significantly upregulated in melanoma tissues and cell lines, and its high expression has been linked to worse OS of melanoma patients. Knockdown of CCAT1 decreases cell proliferation and invasion, induces apoptosis and G_0_/G_1_ cell cycle arrest in melanoma cell lines and inhibits tumor growth in a mouse xenograft model. Mechanistically, CCAT1 acts as a sponge for miR-33a [110] and miR-296-3p [111]. miR-33a is a well-known tumor suppressor downregulated in melanoma that targets snail family zinc finger 2 (SNAI2) and hypoxia inducible factor 1 (HIF-1) transcription factors positively regulating EMT and glycolysis, respectively [129,161], and tumor-promoting cyclin dependent kinase 16 (CDK16) [162]. miR-296-3p targets integrin subunit alpha 9 (ITGA9), an extracellular matrix component that is involved in positive regulation of cell adhesion, migration and invasion [163]. miR-33a is also a target of LINC00518 [129]. This lncRNA is overexpressed in melanoma and triggers radioresistance by regulating glycolysis through miR-33a-3p/HIF-1α axis. In addition, LINC00518 promotes the invasion and migration of melanoma cells through regulating Adaptor Related Protein Complex 1 Sigma 2 Subunit (AP1S2), by binding miR-204-5p, that targets AP1S2. LINC00518 is significantly upregulated in melanoma tissue, and high LINC00518 level may be used as an independent risk factor for the prognosis of melanoma patients [130].

The expression of H19 imprinted maternally expressed transcript lncRNA is increased in melanoma tissues and cell lines and high H19 level has been associated with advanced tumor invasion and TNM stage, distal and lymph node metastases and shorter OS in patients with melanoma. [121,164]. Knockdown of H19 inhibits phosphatidylinositol-4,5-bisphosphate 3-kinase catalytic subunit alpha (PI3K)/AKT serine/threonine kinase 1 and nuclear factor kappa B subunit 1 (NF-κB) signaling, reverses EMT-like process in melanoma cell lines and suppresses tumor growth in immunocompromised mice with melanoma [164,165]. Moreover, H19 functions as a sponge for miR-106a-5p, a tumor suppressor miRNA that targets E2F3 [121], and miR-18b that targets IGF1 [122] H19-mediated upregulation of E2F3 promotes glucose metabolism and growth of melanoma cells [121]. This lncRNA is involved in the regulation of glucose metabolism also in other cancers such as ovarian cancer [166]. Sponging miR-18b by H19 is involved in resistance of melanoma cells to cisplatin [122]

Hox transcript intergenic antisense RNA (HOTAIR) gene is located on chromosome 12q13.13 between HOXC11 and HOXC12, and this lncRNA has been identified as an element of PRC2-dependent gene silencing machinery [167]. HOTAIR has been related to the development of several solid cancers, such as breast cancer and melanoma, where it modulates cancer initiation, progression and drug resistance [168]. HOTAIR is overexpressed in primary and metastatic melanoma lesions, when compared to benign melanocytic nevi. Knockdown of HOTAIR suppresses motility of metastatic melanoma cell lines, which is manifested by decreased gelatinase activity of MMP2 and MMP9 [169]. This lncRNA sponges miR-152-3p which in turn targets c-MET [123]. Moreover, high HOTAIR expression has been also found in intratumoral lymphocytes, and its expression has been correlated with the distance from the tumor site, suggesting that HOTAIR might influence melanoma immunogenicity. HOTAIR has been also identified in serum of melanoma patients, which indicates that its levels might potentially serve as a marker of melanoma incidence [170].

Non-coding RNA activated by DNA damage (NORAD, also known as LINC00657), a highly conserved lncRNA in mammals, is significantly upregulated in melanocytes treated with UVB [171]. In melanoma, its upregulation triggers overexpression of prolyl hydrolase egl-9 family hypoxia inducible factor 2 (EGLN2) through sponging miR-205, a direct target of this transcript [144]. EGLN2 is a regulator of endoplasmic reticulum (ER) stress, and although the role of ER stress in cancer is context-dependent [172], the inhibition of ER stress through NORAD knockdown decreases invasion and migration of melanoma cell lines [144].

lncRNA forkhead box D3 antisense RNA 1 (FOXD3-AS1) is overexpressed in melanoma and functions as oncogene by sponging miR-127-3p and miR-325 and upregulating their target genes four-jointed box kinase 1 (FJX1) and mitogen-activated protein kinase kinase kinase 2 (MAP3K2), respectively [119,120]. Although both genes have not been extensively studied in melanoma so far, MAP3K2 participates in regulation of several pathways such as MAPK signaling, β-catenin pathway and Hedgehog (Hh) pathway in medulloblastoma and osteoblasts [173,174], and FJX1 promotes angiogenesis in colorectal carcinoma [175]. Both genes are upregulated in melanoma and promote proliferation, invasion and migration.

LINC00520 has been found to be overexpressed in melanoma tissues. The high expression of LINC00520 is closely related to the clinical stage of melanoma and survival rate of melanoma patients with high LINC00520 levels is lower than survival rate of patients with its low expression [176]. LINC00520 exerts its oncogenic role by sponging miR-125b-5p to promote eukaryotic initiation factor 5A2 (EIF5A2) expression. Upregulated EIF5A2, a downstream target of PI3K/Akt, promotes melanoma cell proliferation, invasion and migration [127].

The expression of LINC01158 is elevated in melanoma cells resistant to dacarbazine. This lncRNA directly sponges miR-650 to positively regulate the level of O^6^-methylguanine-DNA-methyltransferase (MGMT) oncogene in melanoma cells [132].

Lipoma HMGIC fusion partner-like 3 antisense 1 (LHFPL3-AS1) expression is markedly elevated in melanoma tissues and cells and its high expression in melanoma patients has been associated with TNM stage and distant metastasis. LHFPL3-AS1 can be transcriptionally activated by signal transducer and activator of transcription 3 (STAT3) and this lncRNA provides STAT3 positive feedback loop by sponging STAT3-targeting miR-580-3p [125]. Accordingly, overexpression of LHFPL3-AS1 promotes growth and invasion of melanoma cells. Moreover, LHFPL3-AS1 suppresses apoptosis by sponging miR-181a-5p that targets anti-apoptotic B-cell CLL/lymphoma 2 (BCL2) [126]. Moreover, LHFPL3-AS1-dependent inhibition of BCL2 degradation maintains the stemness of melanoma cells [126].

MIR155HG (miR-155 host gene) is upregulated in melanoma cell lines and tissues and its elevated level has been linked to decreased OS in melanoma patients [139]. SP1 transcription factor is responsible for MIR155HG upregulation in melanoma. MIR155HG knockdown impairs melanoma cells proliferation, migration and invasiveness. Mechanistically, MIR155HG sponges miR-485-3p that binds PC4 and SFRS1 interacting protein 1 (PSIP1) oncogene [139].

MIR205HG (miR-205 host gene) has been significantly upregulated in several melanoma cell lines, when compared to normal human melanocytes, and in patient-derived melanoma tissues, when compared to normal adjacent skin tissue. According to TCGA database, high expression of MIR205HG has been associated with lower survival rate of melanoma patients. miR-299-3p, a tumor suppressor miRNA binds MIR205HG [140]. Knockdown of MIR205HG significantly increases apoptosis and reduces migration and invasion of melanoma cells, and these effects are reversed after the treatment with miR-299-3p inhibitor [140].

ZEB1 antisense RNA 1 (ZEB1-AS1) is located in the opposite strand of gene Zinc Finger E-Box Binding Homeobox 1 (ZEB1). Transcription of both ZEB1-AS1 and ZEB1 is positively correlated in primary melanoma cells, when compared to melanocytes, and in metastatic melanoma cells, when compared to primary melanoma cells [177]. Apart from upregulating ZEB1, ZEB1-AS1 is suggested to sponge miR-1224-5p, which has been identified as a tumor suppressor in malignant gliomas. Knockdown of ZEB1-AS1 or transfection with miR-1224-5p suppresses melanoma cell proliferation, migration and invasion [151]. Interestingly, ZEB1-AS1 expression is significantly higher in BRAF- or RAS-mutated melanomas, when compared to triple-negative (BRAF^WT^, NRAS^WT^, NF1^WT^) melanoma cells, whereas ZEB1 expression is higher only in BRAF^mut^ melanomas [177]. ZEB1-AS1/ZEB1 expression is positively correlated with an invasive phenotype and negatively correlated with a proliferative phenotype of both primary and metastatic melanomas. [177]. Moreover, high expression of ZEB1-AS1 is correlated with decreased OS in melanoma patients [151].

KCNQ1OT1 (KCNQ1 overlapping transcript 1) expression is upregulated in melanoma tissues and cells. KCNQ1OT1 promotes cell proliferation and metastasis in melanoma by sponging miR-153, which results in the upregulation of MET oncogene [124]. MET inhibition decreases AKT phosphorylation, tumor cell proliferation, migration, and induces apoptosis. The combination of vemurafenib and MET-targeting siRNA could inhibit cell growth and reduce cell invasion and migration by melanoma cells with MET amplification [178].

Nuclear enriched abundant transcript 1 (NEAT1) is positively regulated by epidermal growth factor receptor (EGFR) pathway in glioblastoma and its expression contributes to cancer progression mostly via EZH2-dependent repression of several tumor suppressor genes, such as Axin2, inhibitor of beta-catenin and Tcf-4 (ICAT) and glycogen synthase kinase 3 beta (GSK3B) [179]. In melanoma, however, this highly expressed lncRNA mostly acts as a sponge for miR-23a-3p [141], miR-200b-3p [142] and miR-495-3p [143]. This activity results in upregulation of Kruppel-like factor 3 (KLF3), SMAD family member 2 (SMAD2) and E2F3 oncogenes, respectively. Overexpressed KLF3 and E2F3 increased melanoma proliferation, migration and invasion [141,180]. Finally, activated transforming growth factor beta (TGFβ)-SMAD2 pathway is involved in melanocytic oncogenic progression and is responsible, at least in part, for the switch from radial to vertical growth of melanoma [181].

Melanoma Highly Expressed Noncoding RNA (MHENCR) is upregulated in melanoma tissues and further upregulated in metastatic melanoma cell lines. Increased expression of MHENCR indicates lower survival of melanoma patients when compared to patients with its low expression [138]. MHENCR knockdown significantly inhibits melanoma proliferation and migration and induces cell cycle arrest and apoptosis. MHENCR specifically binds miR-425 and miR-489 and in turn upregulates their target genes insulin-like growth factor 1 (IGF1) and spindlin 1 (SPIN1), respectively. The expression of both genes further activates PI3K/AKT pathway [138].

The lncRNA X-inactive specific transcript (XIST) is overexpressed in melanoma and regulates the following miRNA-mRNA axes: miR-23a-3p/GINS complex subunit 2 (GINS2) [149] and 139-5p/Rho associated coiled-coil containing protein kinase 1 (ROCK1) [150]. Both genes are upregulated in melanoma and are involved in positive regulation of melanoma cells proliferation, migration and invasion [149,150].

Overexpressed taurine upregulated 1 (TUG1) might serve as a prognostic marker of poor prognosis for melanoma patients. TUG1-mediated sponging of miR-129-5p induces astrocyte elevated gene (AEG1), which promotes PI3K/AKT and Wnt pathways [145]. Moreover, TUG1 also regulates miR-29c-3p/regulator of G-protein signaling 1 (RGS1) axis, promoting AKT and ERK activation [146].

lncRNAs described above act as tumor-promoting molecules. However, there are several lncRNAs that act as tumor-suppressive miRNA decoy molecules and whose expression is lowered in melanoma compared to normal samples. One of them is maternally expressed gene 3 (MEG3), which suppresses melanoma proliferation, has a pro-apoptotic activity and its low expression is correlated with poor prognosis for melanoma patients [136,137,182]. MEG3 can directly regulate the following axes: miR-499-5p/CYLD lysine 63 deubiquitinase [136] and miR-21/E-cadherin, [137]. CYLD is a negative regulator of various oncogenic pathways including NF-κB [183] and TGFβ [184], and E-cadherin enhances cell-to-cell interactions thereby preventing metastases [185]. Moreover, MEG3 forced overexpression inactivates Wnt signaling by downregulating β-catenin and Cyclin D1 and up-regulating GSK3β protein expression [182].

The expression of cancer susceptibility candidate 2 CASC2 has been downregulated in malignant melanoma tissues and cell lines. This lncRNA sponges miR-18a-5p and miR-181a, which in turn promotes upregulation of RUNX family transcription factor 1 and plexin C1 (PLXNC1), respectively [108,109]. Both genes are downregulated in melanoma, when compared to normal melanocytes, and their re-expression inhibits melanoma proliferation [108,186]. LINC00961 inhibits cell proliferation and promotes apoptosis by upregulation of phosphatase and tensin homolog (PTEN) melanoma tumor suppressor, which is mediated by sponging PTEN-targeting miR-367 [131].

LINC00459 downregulated in melanoma sponges miR-218 which in turn downregulates dickkopf WNT signaling pathway inhibitor 3 (DKK3) [128]. DKK3 expression is usually lost at early stages of melanoma development [187]. LINC00459 low expression is correlated with decreased patient survival rates [128].

### 3.2. circRNAs as miRNA Sponges in Melanoma

circRNA_0084043 is one of the first circular RNAs identified in melanoma. Its level is higher in melanoma tissues and cell lines than in normal cells, and its high expression has been correlated with clinical stage of melanoma and decreased OS of melanoma patients [117]. circRNA_0084043 has a sponging activity for miR-153-3p, that functions as a melanoma tumor suppressor by targeting Snai1 transcription factor responsible for promoting melanoma cells proliferation and invasiveness [188]. Migration and invasion of melanoma cells are significantly reduced after circRNA_0084043 knockdown [117]. In addition to miR-153-3p sponging and controlling EMT, circRNA_0084043 has been also found to sponge another tumor suppressor miR-429, which negatively regulates Wnt/β-catenin pathway through binding to tribbles pseudokinase 2 (TRIB2) [118], a downstream effector gene of TCF transcription factors signaling [189].

circ_0002770, produced from exons 8, 9 and 10 of MDM2 proto-oncogene, is also overexpressed in melanoma cell lines and tissues [112]. Melanoma patients with high expression of circ_0002770 are characterized with shorter survival time than those with its lower expression. circ_0002770 knockdown significantly decreases migration and colony formation of melanoma cell lines and inhibits tumor growth in mouse xenograft. circ_0002770 exerts its tumor-promoting role by sponging miR-331-3p, which in turn promotes expression of MAPK pathway regulators dual specificity phosphatase 5 (DUSP5) and transforming growth factor beta receptor 1 (TGFBR1) [112].

circ_0079593 is another circular RNA significantly overexpressed in human melanoma tissues and cell lines [116]. Patients in low circ_0079593 group have a longer survival time than those in high circ_0079593 group. Moreover, the expression of circ_0079593 has been associated with melanoma clinical stage and Breslow thickness. siRNA-mediated circ_0079593 knockdown represses cell proliferation, colony formation, cell cycle progression, migration, invasion, and enhances apoptosis in vitro. circ_0079593 knockdown is accompanied by significant decrease in oncogenic abhydrolase domain containing 2, acylglycerol lipase (ABHD2) and increased level of miR-573. ABHD2 is a direct target of miR-573, which in turn is sponged by circ_0079593 in melanoma cell lines [116].

circ_0020710 is derived from back-splicing of CD151 transcript and resides mostly in cytoplasm. Both CD151 and CD151-derived circ_0020710 have been overexpressed in melanoma tissues when compared to control samples. circ_0020710 expression has been significantly correlated with the advanced Breslow depth and Clark level. Patients with high circ_0020710 expression are characterized with shorter OS than those with low circ_0020710 expression [114]. Mechanistically, circ_0020710 sponges miR-370-3p which in turn targets C-X-C motif chemokine ligand 12 (CXCL12).

circ_0025039, a circRNA transcript formed by exons 4 and 5 of the FOXM1 gene on chromosome 12, is elevated in melanoma tissues and cell lines, and promotes cell growth, invasion and glucose metabolism [115]. Melanoma patients with high circ_0025039 expression are characterized with shorter survival time. This circRNA sponges miR-198 which negatively regulates CDK4 expression, and circ_0025039 silencing leads to decreased tumor formation in vivo [115]. circ_0016418 is upregulated in melanoma cells and its overexpression suppressed proliferation, migration, invasion, and EMT-like process. circ_0016418 exerts its oncogenic properties by sponging miR-625, which in turn binds yin-yang 1 (YY1) transcription factor [113]. YY1 acts upstream MITF and cMYC pathways and governs multiple metabolic pathways and protein synthesis in neural crest stem cells and melanoma [190]. It has been found that metabolic programming that accompanies transition from melanocytes to melanoma cells is, at least partially, dependent on YY1 expression and that YY1 knockdown is sufficient to prevent melanocyte transformation [190].

### 3.3. The ceRNA Controversy

Although the notion that lncRNAs and circRNAs can act as effective ceRNAs is widely recognized, several authors question the relevance of sponging activity of these RNA molecules in natural physiological setting (revised in depth in [191]). Denzler et al., working with human hepatocytes that produce high level of miR-122, one of the most abundant miRNA in human tissues, have proved that highly-expressed miRNAs are immune to decreased activity triggered by ceRNAs, even when overexpressed, which results in lack of miRNA targets de-repression [192]. This observation has been further broadened to other miRNAs and cell types, e.g., let-7, miR-194, and miR-192 in human embryonic stem cells [193]. The most important conclusions from these experiments are that: (I) usually, only lowly expressed miRNA might be prone to ceRNA-mediated miRNA target de-repression, (II) the ceRNA-mediated miRNA target de-repression is generally dependent on the relative stoichiometric between ceRNAs and miRNAs, and (III) physiological changes in ceRNAs levels are not sufficient to remarkably outcompete in miRNAs binding to their targets. However, a novel mode of lncRNA-dependent miRNA degradation has been discovered recently, named target-directed miRNA degradation (TDMD). Extended base pairing between lncRNAs and miRNAs can expose the 3′ end of miRNA bound to argonaute (AGO) protein to ubiquitin-mediated proteolysis resulting in miRNA degradation [194,195]. For instance, lncRNA CYRANO mediates miR-7 degradation in nervous cells through TDMD [196].

Other authors also argue about the sponging properties of circRNAs. With several exceptions, such as for CDR1-AS1, which harbors 63 conserved miR-7 target sites and indeed regulates miR-7 abundance and activity in central nervous system [87], the vast majority of circRNAs do not harbor such distinct and conserved MREs [104,197,198], therefore their sponging activity is substantially limited. Moreover, due to their circular nature and specific mechanism of expression and splicing, circRNA overexpression and knockdown experiments, especially in an in vivo setting, are still extremely challenging [199].

However, one should take into consideration several other issues. During carcinogenesis the cellular homeostasis is disturbed with exceptional transcriptome changes that might interfere with the ceRNAs-miRNAs stoichiometric relationship and leads to dysregulation of the whole cellular ceRNA network [200]. Moreover, despite low overall expression levels of the vast majority of lncRNAs, their high compartmentalization or specific subcellular localization might substantially increase their concentration at the site of activity [201]. The expression of lncRNA is also temporally regulated, which suggests that the temporal coordination of lncRNAs expression enhances their sponging activity towards specific target miRNAs [202,203]. Furthermore, the stability of endogenous transcripts varies significantly: lncRNAs secondary structure usually contains several hairpin structures that increase their stability [204], and circRNAs lack 5′ and 3′ ends which makes them highly resistant to exonucleases [86,87]. Their prolonged stability compared to other endogenous RNA molecules might be also crucial in terms of their ceRNAs activity. Moreover, lncRNAs and circRNAs are selectively sorted and released in exosomes from tumor cells [205,206], therefore, their expression levels can be measured with liquid biopsies. Finally, successful knockdown of specific lncRNAs or circRNAs, and introduction of exogenous modified oligonucleotides with sponging activity might lead to desired anti-tumor outcomes, both in vitro and in vivo, as discussed above in this chapter.

## 4. lncRNAs Involved in Melanoma Drug Resistance and Treatment Response

Several recent findings suggest that lncRNAs might also influence the response of melanoma cells to chemotherapy, targeted therapies and immunotherapy and control drug resistance. Knockdown of lncRNA H19 sensitizes cisplatin (DDP)-resistant melanoma cell lines to this drug. H19 silencing inhibits colony formation and promotes apoptosis of cisplatin-resistant melanoma cells, which is abrogated by miR-18b inhibition or IGF1 upregulation [122].

The expression of LINC01158 is elevated in melanoma cells resistant to dacarbazine (DTIC). Overexpression of LINC01158 allows melanoma cells to survive DTIC treatment, while LINC01158 knockdown restores cell sensitivity to this drug [132]. Inhibition of TUG1, overexpressed in melanoma, reduces in vivo tumor growth and increases the chemosensitivity of human melanoma cell lines to DDP and 5-fluorouracil (5-FU) in vitro [145].

The upregulation of XIST is a prognostic marker of resistance to oxaliplatin treatment [207]. This platinium-based chemotherapeutic drug has lower toxicity than cisplatin, and is active in electrochemotherapy in murine melanoma models [208]. Its activity in melanoma patients have been recently under investigation in several clinical trials.

In a retrospective clinical study performed by Kolenda et al., the expression of 90 lncRNA with potential inclinations for cancer development and progression have been examined in plasma samples collected from patients with BRAF-mutated melanoma treated with vemurafenib [209]. It has been demonstrated that three lncRNAs, IGF2 antisense (IGF2AS), MEG3, and zinc finger AE-binding homeobox 2-natural antisense transcript (Zeb2NAT) might serve as prognostic markers of response to vemurafenib treatment in melanoma patients [209].

RMEL3 has been identified as a melanoma-restricted lncRNA whose expression was positively correlated with BRAF^V600E^ and NRAS^Q61L^ mutations [210,211]. This lncRNA is a positive regulator of PI3K and MAPK signaling in melanoma, since its knockdown increases PTEN levels and decreases activated AKT, RAF and ERK levels [211,212]. RMEL3 knockdown significantly decreases colony formation capacity in BRAF^V600E^ melanoma cell lines. Moreover, the UV mutational signature (C > T substitutions in dipyrimidine sites, including CC > TT) is present in more than 70% of analyzed sequences of RMEL3 deposited in TCGA. These mutations are not correlated with RMEL3 expression, however, they have been associated with poor patient survival rates [211]. Finally, BRAF^V600E^ inhibitor vemurafenib significantly downregulates RMEL3 expression, which has been accompanied by FOXD3 induction and decrease in ERK phosphorylation [211].

TLSCN8 lncRNA is significantly downregulated in melanoma cells with acquired resistance to BRAF^V600E^ inhibitor PLX4720. Moreover, downregulation of TSLNC8 in BRAF inhibitor-sensitive melanoma cells inhibits apoptosis after treatment with PLX4720 [58].

MIRAT is overexpressed in several NRAS-mutated melanoma cell lines resistant to MEK inhibitor trametinib, and its expression increases in a time- and dose-dependent manner upon trametinib treatment [51].

The upregulation of EMICERI (EQTN MOB3B IFNK C9orf72 enhancer RNA I) lncRNA promotes the expression of MOB kinase activator 3B (MOB3B) in melanoma cells. MOB3B is a paralog of MOB1A/B kinases, positive regulators of the Hippo signaling pathway, whose activation confers resistance to vemurafenib [213]. MOB3B overexpression downregulates large tumor suppressor kinase 1 (LATS1) to activate the Hippo signaling pathway [214].

Several reports show that various lncRNAs, such as XIST, NEAT1 or NKILA, are generally involved in the regulation of human immune system and tumor-immune cell infiltration [215,216,217]. The SKCM dataset from TCGA, that comprises molecular and clinical data for 470 melanoma patients has been used recently to identify immune-related lncRNAs [218,219]. Ping et al. have applied a modified least absolute shrinkage and selection operator (LASSO) regression model to identify 28 immune-related lncRNAs in melanoma patients, and among them, 17 pairs of co-expressed lncRNAs that could divide SKCM cohort into high-risk and low-risk groups [218]. For instance, the co-expression of MIR205HG and U62631.1 lncRNA pair is correlated with high risk factor (*p*-value < 0.001) and the co-expression of major histocompatibility complex, class II, DQ beta 1 antisense 1 (HLA-DQB1-AS1) and ubiquitin-like modifier-activating enzyme 6 antisense 1 (UBA6-AS1) is linked to the protective effects (*p*-value < 0.001) in SKCM model. Moreover, the high-risk group is correlated with the expression of specific mutant genes such as BRAF and KIT (*p*-value < 0.01) [218]. Wang et al. have used survival analysis and Cox regression model to identify 8 immune-related lncRNAs with prognostic value in SKCM dataset [219]. Again, MIR205HG expression is linked to the poor outcome (*p*-value < 0.001), and HLA-DQB1-AS1 to immune protection (*p*-value = 0.048). Although the results in both reports are convergent, these two computational approaches have limitations: the TCGA SKCM raw data is insufficient to draw conclusions regarding novel melanoma biomarkers and future clinical studies with high amount of melanoma patients should be performed.

Finally, the expression of MIR155HG is positively linked to the expression of immune checkpoint genes such as PD-1, CTLA4, LAG3, and TIM3 in adenocarcinoma and melanoma [220], and elevated circ_0020710 expression, and hence CXCL12 upregulation, is correlated with cytotoxic lymphocyte exhaustion and resistance to anti-PD-1 melanoma treatment [114]. Therefore, high expression of both MIR155HG and circ_0020710 might compromise melanoma immunotherapies based on immune checkpoint inhibitors.

Several lncRNAs might be also considered as direct targets in melanoma therapy. Intravenous and intra-tumor injections of SAMMSON antisense nucleotides in mice resulted in significant tumor growth suppression. Moreover, tumor regression has been observed in mice upon exposure to both dabrafenib and SAMMSON antisense nucleotides, without any relevant adverse effects or weight loss, in contrast to mice treated with a combination of dabrafenib and trametinib [53]. These findings raise the possibility to use SAMMSON as a biomarker of malignancy and highly selective anti-melanoma therapeutic target. Moreover, knockdown of SAMMSON significantly reduces clonogenicity of all SAMMSON-expressing melanoma cells independently of their BRAF, NRAS or TP53 status, and enhances the cytotoxic effect of vemurafenib and MEK inhibitor pimasertib in drug resistant cell lines [53]. Moreover, SOX10-mediated transcriptional induction of SAMMSON by vemurafenib might be one of the mechanisms that confer resistance of melanoma cells to targeted therapies against MAPK pathway. SAMMSON knockout sensitized BRAF-mutated melanoma to BRAF inhibitors in vitro and in vivo, and induced p53 signaling [221].

Specific antisense oligonucleotides (ASO) have been used in different solid tumors such as breast, lung, prostate or ovarian cancers and melanoma to target mitochondria-derived nuclear lncRNAs, such as antisense non-coding mitochodrial RNAs ASncmtRNA-1 and ASncmtRNA-2 [222]. Knockdown of both non-coding mitochondrial RNAs by ASO triggers inhibition of cell proliferation and induces apoptosis in murine melanoma in vitro and in vivo models [223,224]. One antisense oligonucleotide drug named Andes-1537 has been included in two clinical trials in advanced solid tumors such as cervical, gastric and pancreatic cancers: NCT02508441 (terminated) and NCT03985072 (recruiting).

Another treatment approach involving lncRNAs comprises the use of natural plant secondary metabolites (phytochemicals) with proven anti-tumor activity, such as curcumin, genistein, quercetin or resveratrol. These and other compounds have been found to affect the expression of cancer-related lncRNAs and might be regarded as promising anti-cancer drugs [225].

## 5. Conclusions and Future Perspectives

Despite recent advances in treatment and development of inhibitors that selectively target mutated BRAF and other elements of the MAPK pathway in melanoma, or inhibitors of immune checkpoints PD-1 and CTLA-4, low response rate or acquired resistance to these drugs remain the main cause of melanoma recurrence and patient deaths. The findings reviewed in this paper indicate that lncRNAs are novel emerging regulatory molecules that: (I) affect melanoma proliferation, invasion, migration and apoptosis, (II) are directly involved in melanoma development and may promote drug resistance, and (III) might be used as prognostic biomarkers of melanoma in the future (Table 3). And finally, tissue-specific expression of lncRNAs potentiates them as therapeutic targets [226], especially if lncRNA is overexpressed in cancer cell type, including melanoma [53].

Recent advances in RNA-sequencing methods allow identification of novel lncRNAs, or new unannotated splicing variants and advanced bioinformatic tools predict lncRNA interactions with other molecules and thus suggest their function in cancers [39]. Many free online databases and programs can help to predict the function of identified lncRNAs or circRNAs in cancer development and progression. These include AnnoLnc2 for human lncRNAs annotation [229], LNCipedia, a comprehensive compendium of human long non-coding RNAs [230], LncExpDB, an expression database of human lncRNA genes [231], LincSNP 3.0, a database for single nucleotide polymorphisms (SNPs) in human lncRNAs [232], lnCAR, a comprehensive resource for lncRNAs in cancers [233], or databases for circular RNAs such as circBASE [234] or circInteractome [235]. Sun et al. have identified 246 differentially-expressed lncRNAs in metastatic melanoma cell lines when compared to primary melanomas [236]. Among them, 14 lncRNAs have been associated with increased or decreased OS of melanoma patients, which makes them potential biomarkers of disease prognosis. Finally, the International Cancer Genome Consortium (ICGC)/The Cancer Genome Atlas (TCGA) Pan-Cancer Analysis of Whole Genomes Consortium have identified a differential and peculiar transcriptional signatures between melanoma and normal tissues [237]. Subsequent validation of predicted interactions can be achieved with experimental methods such as RNA pull-down assay, RNA immunoprecipitation (RIP) [238] or high-throughput sequencing of RNA isolated by crosslinking and precipitation (HITS-CLIP) and photoactivatable ribonucleoside-enhanced crosslinking and immunoprecipitation (PAR-CLIP) [239]. Finally, the experimental validation of lncRNA function in cancer, with the use of CRISPR/CAS9 genetic modification is needed [240].

Taken together, lncRNAs may be considered as novel emerging molecules that have important regulatory functions in melanomagenesis and regarded as potential biomarkers or therapeutic targets in melanoma. However, this approach has some limitations for the time being, and future large-scale research needs to be performed to verify in vitro and in vivo results in clinical settings. One of the challenges with the clinical application of lncRNAs as cancer biomarkers is how to develop convenient and quick techniques to detect target lncRNAs in melanoma patients, taking into consideration their generally low expression in cells and tissues. Another severe limitation is that reported results for some lncRNAs are conflicting. For instance, the increased expression of HOTAIR has been identified in melanoma samples compared to normal tissues by Tang et al. [169], whereas Tian et al. [155] did not find any significant difference between the HOTAIR expression in primary melanoma samples and adjacent normal tissues. Moreover, a number of analyzed samples might be insufficient to draw reliable conclusions or selected cell lines might not represent all subsets of melanomas identified in patients. Still little is known about all lncRNA interactions in the human body, therefore it is difficult to design drug that would specifically target particular lncRNAs without possible off-target effects. Thus, using lncRNAs as potential druggable targets might introduce adverse and toxic effects to the patients.

Finally, observed interactions need additional and independent confirmation in adequately-powered experiments to choose proper lncRNAs and use them as melanoma biomarkers or druggable target molecules in the future. So far, lncRNA-related clinical trials in diverse solid cancers, such as hepatocellular, colorectal or breast cancers, are focused on the identification of diagnostic values of lncRNAs and their relation to tumor staging (observational studies). Recent (not yet recruiting) interventional clinical trial (NCT04946968) for EGFR-driven solid tumors, such as non-small cell lung carcinoma and head and neck squamous cell carcinoma with low level of lncRNA EGFR-AS1, has been established to investigate the response to EGF-1R inhibitor dacomitinib. However, to our knowledge, no clinical trials assessing lncRNA expression in melanoma have been so far developed.

## Figures and Tables

**Table 1:** Protein-lncRNA interactions in melanoma.

lncRNA	Expression in Melanoma ^a^	Biding Partner (s)	The Effect of lncRNA-Protein(s) Interaction	Mode of Action (in cis/in trans)	References
ANRIL and circANRIL	upregulated	CBX7 (PRC1)	ANRIL binds to PRC1 and represses the expression of the CDKN2A/B locus	in cis	[42]
CASC15	upregulated	EZH2	CASC15 binds to EZH2 and together silence the expression of PDCD4 tumor suppressor gene	in trans	[43]
CDR1as	downregulated	IGF2BP3	CDR1as inhibits pro-invasive properties of IGF2BP3 which are unleashed after CDR1as loss	N/A	[44]
DIRC3	downregulated	SOX10	acts locally to block SOX10 chromatin binding at melanoma regulatory elements and activate IGFBP5 expression	in cis	[45]
FALEC	upregulated	EZH2	FALEC-EZH2 complex binds to p21 promoter and inhibits p21 expression	in trans	[46]
GAS5	downregulated	E2F4	GAS5 recruits E2F4 transcription factor to EZH2 promoter, which represses EZH2 expression	in trans	[47]
HEIH	upregulated	EZH2	HEIH-EZH2 repressive complex binds to the promoter of miR-200 family tumor suppressor miRNAs	in trans	[48]
LNMAT1	upregulated	EZH2	LNMAT1-EZH2 binds to CADM1 promoter and inhibits expression of this tumor suppressor gene	in trans	[49]
MIR31HG	upregulated	SUZ12 and EZH2	MIR31HG binds to PRC2 represses the expression of p16 tumor suppressor gene	in cis	[50]
MIRAT	upregulated	IQGAP1	MIRAT binds to and stabilizes IQGAP1, which promotes MAPK signaling	N/A	[51]
PVT1	upregulated	EZH2	PVT1-EZH2 binds to miR-200c promoter and inhibits expression of this miRNA	in trans	[52]
SAMMSON	upregulated	p32	SAMMSON stabilizes the mitochondrial protein p32, required for oxidative phosphorylation	N/A	[53]
SLNCR1	upregulated	Brn3a and AR AR and EGR1	binds to MMP9 promoter and activates MMP9 transcription represses EGR1-inducible expression of p21	in trans in trans	[54] [55]
SPRY4-IT1/SPRIGHTLY	upregulated	lipin 2	SPRIGHTLY binding to lipin 2 blocks its phosphatidic acid phosphatase activity	N/A	[56]
THOR	upregulated	IGF2BP1	directly binds to and stabilizes IGF2BP1; this interaction promotes melanoma growth	in trans	[57]
TSLNC8	upregulated	PP1α	TSLNC8 triggers PP1α accumulation in cytoplasm which results in reactivation of MAPK signaling	N/A	[58]
TTN-AS1	upregulated	TTN	High expression of TTN-AS1 and TTN induces melanoma proliferation, and suppresses cell apoptosis,	N/A	[59]

^a^ compared to healthy tissues and normal cells; N/A, not applicable.

**Table 2:** lncRNA and circRNAs with miRNA sponging activity in melanoma.

lncRNA	Function	Expression in Melanoma ^a^	miRNA-mRNA Axis	References
BANCR	oncogene	upregulated	miR-204-5p/notch2	[107]
CASC2	tumor suppressor	downregulated	miR-18a-5p/RUNX1 miR-181a/PLXNC1	[108] [109]
CCAT1	oncogene	upregulated	miR-33a/N/A miR-296-3p/ITGA9	[110] [111]
circ_0002770	oncogene	upregulated	miR-331-3p/DUSP5, TGFBR1	[112]
circ_0016418	oncogene	upregulated	miR-625/YY1	[113]
circ_0020710	oncogene	upregulated	miR-370-3p/CXCL12	[114]
circ_0025039	oncogene	upregulated	miR-198/CDK4	[115]
circ_0079593	oncogene	upregulated	miR-573/ABHD2	[116]
circ_0084043	oncogene	upregulated	miR-153-3p/Snail miR-429/TRIB2	[117] [118]
FOXD3-AS1	oncogene	upregulated	miR-127-3p/FJX1 miR-325/MAP3K2	[119] [120]
H19	oncogene	upregulated	miR-106a-5p/E2F3 miR-18b/IGF1	[121] [122]
HOTAIR	oncogene	upregulated	miR-152-3p/c-MET	[123]
KCNQ1OT1	oncogene	upregulated	miR-153/MET/MYC	[124]
LHFPL3-AS1	oncogene	upregulated	miR-580-3p/STAT3 miR-181a-5p/BCL2	[125] [126]
LINC00520	oncogene	upregulated	miR-125b-5p/EIF5A2	[127]
LINC00459	tumor suppressor	downregulated	miR-218/DKK3	[128]
LINC00518	oncogene	upregulated	miR-33a-3p/HIF-1α miR-204-5p/AP1S2	[129] [130]
LINC00961	tumor suppressor	downregulated	miR-367/PTEN	[131]
LINC01158	oncogene	upregulated	miR-650/MGMT	[132]
MALAT1	oncogene	upregulated	miR-34a/c-Myc, MET miR-183/ITGB1 miR-22/MMP14, Snail	[133] [134] [135]
MEG3	tumor suppressor	downregulated	miR-499-5p/CYLD miR-21/E-cadherin	[136] [137]
MHENCR	oncogene	upregulated	miR-425/IGF1 miR-489/SPIN1	[138]
MIR155HG	oncogene	upregulated	miR-485-3p/PSIP1	[139]
MIR205HG	oncogene	upregulated	miR-299-3p/VEGFA	[140]
NEAT1	oncogene	upregulated	miR-23a-3p/KLF3 miR-200b-3p/SMAD2 miR-495-3p/E2F3	[141] [142] [143]
NORAD	oncogene	upregulated	miR-205/EGLN2	[144]
TUG1	oncogene	upregulated	miR-129-5p/AEG1 miR-29c-3p/RGS1	[145] [146]
UCA1	oncogene	upregulated	miR-28/HOXB3 miR-507/FOXM1	[147] [148]
XIST	oncogene	upregulated	miR-23a-3p/GINS2 39-5p/ROCK1	[149] [150]
ZEB1-AS1	oncogene	upregulated	miR-1224-5p/N/A	[151]

^a^ compared to healthy tissues and normal cells; N/A, not available.

**Table 3:** lncRNAs as prognostic markers in melanoma.

lncRNA	Expression in Melanoma ^a^	Association with Melanoma Treatment Outcomes	References
BANCR	upregulated	high expression correlated with melanoma stage (*p* = 0.017, *n* = 103), and lower OS (*p* < 0.01, *n* = 72)	[156]
CASC15	upregulated	high expression associated with TNM stage (*p* = 0.013), distal metastasis (*p* = 0.018) and lymphatic metastasis (*p* = 0.006) (*n* = 461)	[43]
CCAT1	upregulated	high expression linked to worse OS of melanoma patients (*p* = 0.038, *n* = 30)	[110]
CDR1as	downregulated	low expression correlated with shorter PFS (0.0008) and OS (*p* = 0.0023) of melanoma patients (*n* = 53)	[44]
circ_0025039	upregulated	patients with high circ_0025039 expression were characterized with shorter OS (*p* < 0.05, *n* = 43)	[115]
circ_0002770	upregulated	high expression correlated with shorter OS of melanoma patients (*p* < 0.05, *n* = 20)	[112]
circ_0020710	upregulated	expression correlated with the advanced Breslow depth (*p* = 0.012) and Clark level (*p* = 0.034); high expression characterized with shorter OS (*p* = 0.036) in melanoma patients (*n* = 88)	[114]
circ_0079593	upregulated	low expression correlated with longer survival time (*p* = 0.004); expression associated with melanoma clinical stage (*p* = 0.028) and Breslow thickness (*p* = 0.014) of melanoma patients (*n* = 47)	[116]
circ_0084043	upregulated	high expression correlated with clinical stage of melanoma (*p* < 0.01, *n* = 30) and decreased OS of melanoma patients (*p* < 0.05, *n* = 33)	[117]
FALEC	upregulated	high expression linked to poorer patients OS (*p* < 0.001) and TNM stage (*p* = 0.012) of melanoma patients (*n* = 78)	[46]
GAS5	downregulated	low expression correlated with the TNM staging of melanoma patients (*p* < 0.05, *n* = 94)	[74]
H19	upregulated	high expression associated with advanced tumor invasion and TNM stage (*p* < 0.001), distal (*p* = 0.015) and lymph (*p* = 0.048) node metastases and shorter OS (*p* < 0.05) of melanoma patients (*n* = 30)	[122,164]
HEIH	upregulated	high expression associated with advanced clinical stages; might predict poor clinical outcomes in melanoma patients. (66 patients with melanoma and 42 patients with benign nevi, *p* = 0.026)	[48]
HOTAIR	upregulated	identified in serum of melanoma patients (*n* = 34); correlated with melanoma incidence	[170]
IGF2AS MEG3 Zeb2NAT	downregulated downregulated upregulated	combined expression levels of these three lncRNAs measured in plasma patients might serve as prognostic markers of response to vemurafenib treatment in melanoma patients	[209]
LHFPL3-AS1	upregulated	high expression in melanoma patients (*n* = 52) associated with TNM (*p* = 0.009) stage and distant metastasis (*p* = 0.012)	[125]
LINC00518	upregulated	high expression was an independent risk factor for the prognosis of melanoma patients (OS: *p* = 0.03, *n* = 458)	[130]
LINC00459	downregulated	low expression correlated with decreased OS (*p* = 0.032) and FPS (0.009) of melanoma patients (*n* = 126)	[128]
LINC00520	upregulated	high expression closely related to the clinical stage of melanoma (*p* < 0.01) and OS (*p* < 0.05) of melanoma patients (*n* = 38)	[176]
LINC01550	downregulated	high expression associated with increased OS (*p* = 0.015, *n* = 541) and DFS (*p* = 0.042, *n* = 380) in melanoma patients	[227]
MEG3	downregulated	low expression correlated with poor prognosis for melanoma patients (OS: *p* = 0.001, *n* = 42)	[136]
MHENCR	upregulated	high expression indicated poor OS of melanoma patients (*p* = 0.017, *n* = 30)	[138]
MIR205HG	upregulated	high expression of MIR205HG associated with lower OS of melanoma patients (*p* = 3.5e-6, *n* = 521)	[140]
PVT1	upregulated	high expression negatively correlated with OS (*p* = 0.021) of melanoma patients (*n* = 35).	[228]
RMEL3	upregulated	identified mutations associated with poor patient survival rates (*p* < 0.05, *n* = 38)	[211]
SAMMSON	upregulated	high expression negatively correlated with cytotoxic effects of vemurafenib and pimasertib in melanoma cell lines	[53]
SLNCR1	upregulated	high expression was associated with shorter OS in melanoma patients (*p* = 0.043; *n* = 213)	[54]
SPRIGHTLY	upregulated	identified in plasma of melanoma patients (*n* = 70), associated with tumor stage (*p* < 0.001); high expression significantly reduced OS rates (*p* < 0.001) of melanoma patients	[79]
TTN-AS1	upregulated	high expression correlated with poor OS of melanoma patients (*p* = 0.048, *n* = 165)	[59]
TUG1	upregulated	Overexpressed might serve as a prognostic marker of poor prognosis for melanoma patients (OS: *p* = 0.026, *n* = 48)	[146]
UCA1	upregulated	high expression positively correlated with melanoma stage (*p* = 0.046, *n* = 63)	[155]
ZEB1-AS1	upregulated	high expression is correlated with decreased OS of melanoma patients (*p* = 0.05, *n* = 46).	[151]

^a^ Compared to healthy tissue; OS, overall survival; DFS, disease-free survival; *p*, *p*-value; PFS, progression-free survival; *n*, number of specimens (patients or tissue samples); TNM, tumor, nodes and metastases.
